# Cardiac Medication Use in ACTION for Duchenne Muscular Dystrophy Cardiomyopathy

**DOI:** 10.1007/s00246-025-03917-2

**Published:** 2025-06-20

**Authors:** Carol A. Wittlieb-Weber, Brian F. Birnbaum, Chesney D. Castleberry, Tyler W. Cunningham, Paul Esteso, Katheryn E. Gambetta, Emily A. Hayes, Daphne T. Hsu, Beth D. Kaufman, Benjamin Kroslowitz, Ashwin K. Lal, Angela Lorts, Hugo Martinez, Deepa Mokshagundam, Deipanjan Nandi, John J. Parent, Frank Raucci, Nelia Soares, Jonathan H. Soslow, Renata Shih, Svetlana Shugh, Chet R. Villa, Sarah J. Wilkens, Bethany L. Wisotzkey, Jennifer Conway

**Affiliations:** 1 Perelman School of Medicine at the University of Pennsylvania, Children’s Hospital of Philadelphia, 3401 Civic Center Boulevard, Philadelphia, PA 19104 USA; 2 Children’s Mercy Kansas City, Kansas City, USA; 3 Dell Children’s Medical Center, Austin, USA; 4 Arkansas Children’s Hospital, Little Rock, USA; 5 Boston Children’s Hospital, Boston, USA; 6 Ann & Robert H. Lurie Children’s Hospital of Chicago, Chicago, USA; 7 Nationwide Children’s Hospital, Columbus, USA; 8 Children’s Hospital at Montefiore, New York, USA; 9 Lucile Packard Children’s Hospital Stanford, Palo Alto, USA; 10 Cincinnati Children’s Hospital, Cincinnati, USA; 11 Primary Children’s Hospital, Salt Lake City, USA; 12 St. Louis Children’s Hospital, St Louis, USA; 13 Riley Hospital for Children at IU Health, Indianapolis, USA; 14 Children’s Hospital of Richmond at VCU, Richmond, USA; 15 Children’s Medical Center at Dallas, Dallas, USA; 16 Monroe Carell Jr. Children’s Hospital at Vanderbilt, Nashville, USA; 17 Shands Children’s Hospital, Gainesville, USA; 18 Joe DiMaggio Children’s Hospital, Hollywood, USA; 19 Norton Children’s Hospital, University of Louisville, Louisville, USA; 20 Seattle Children’s Hospital, Seattle, USA; 21 Stollery Children’s Hospital, Edmonton, Canada

## Abstract

This study seeks to understand cardiac medication use in a large cohort of males with Duchenne Muscular Dystrophy (DMD) followed prospectively with focus on current practices and adherence to consensus directed medical therapy (CDMT). DMD patients have been enrolled in the Advanced Cardiac Therapies Improving Outcomes Network (ACTION) Dystrophinopathy Registry since 2021. Cardiac medication use was analyzed at enrollment and most recent follow-up. CDMT was defined as concurrent use of angiotensin-converting-enzyme inhibitor (ACEi)/angiotensin II receptor blocker (ARB) /angiotensin receptor-neprilysin inhibitor (ARNI) plus beta-blocker (BB) plus mineralocorticoid receptor antagonist (MRA). Two hundred sixty-five males with DMD (median age 17.5 (IQR 14.5–21.5) years) were prospectively followed; median follow-up was 11.5 (IQR 6.2–15.6) months. At most recent follow-up, 153 patients (57.7%) had decreased LV systolic function, 67 (25.3% of the cohort) had moderate or severe dysfunction. For patients with moderate or severe dysfunction, CDMT was used for 49/67 (73.1%) at most recent follow-up, similar to 36/51 (70.6%) at enrollment (p = 0.92). Target doses of CDMT were achieved for 27% of males on ACEi/ARB/ARNI, 28% on BB, and 23% on MRA. Initial analysis of a prospective registry of males with DMD showed that ~ 30% of patients with moderate or severe LV dysfunction were not on CDMT at most recent follow-up and the majority did not reach target dosing. Further understanding regarding the optimal combination of cardiac medications for DMD cardiomyopathy is needed, as is a better understanding of the barriers to CDMT optimization given increasing cardiac causes of death for DMD patients.

**Supplementary Information:**

The online version contains supplementary material available at 10.1007/s00246-025-03917-2.

## Introduction

Duchenne muscular dystrophy (DMD), an X-linked recessive disorder caused by a pathologic variant in the gene that encodes dystrophin, is the most common form of childhood muscular dystrophy, with prevalence estimates of 1.02/10,000 males in the Unites States and 4.78/100,000 males worldwide[1, 2]. Untreated, DMD has a predictable clinical course marked by progressive skeletal muscle weakness with loss of ambulation typically by age 12 and death occurring in early adulthood secondary to respiratory or cardiac failure. Cardiac disease in DMD manifests as progressive cardiomyopathy (CM) and/or cardiac arrhythmia with CM incidence increasing with age[3–5]. Analysis of the Muscular Dystrophy Surveillance Tracking and Research Network (MD STARnet) registry showed that the mean age of first abnormal left ventricular (LV) ejection fraction (EF) was 15.2 ± 3.9 years, with an estimated rate of decline in EF of 1.6% per year[6]. As survival, neuromuscular function, and quality of life in DMD are improving due to treatment with glucocorticoids and advances in respiratory care, cardiac disease is increasing as a major cause of death [7–12].

Cardiac muscle lacking functional dystrophin is mechanically weak, and thus, contraction of the cardiac myocytes leads to loss of membrane integrity resulting in a cascade of increased calcium influx into the cell and eventual cell death with fibro-fatty replacement of cardiac muscle[13–15]. In DMD, fibro-fatty replacement of cardiac myocytes follows a clear pattern and has been shown to be a predictor of cardiac remodeling, ventricular arrhythmias, and death [16–18]. Numerous studies have assessed whether the progressive cardiovascular dysfunction of DMD can be altered with oral medications, focusing on standard adult heart failure (HF) medications targeting the renin–angiotensin–aldosterone system and the sympathetic nervous system[19–21]. Unique to the DMD population is the concept of therapeutic verse prophylactic intervention determined by the presence or absence of systolic dysfunction respectively, with the 2018 DMD Care Considerations recommending initiation of an angiotensin-converting enzyme inhibitor (ACEi) by age 10 even in the presence of normal LV systolic function [22].

The basis for prophylactic ACEi use in patients with DMD with preserved LV EF is derived from a trial conducted by Duboc et al., comparing ACEi use to placebo in a cohort of patients with DMD, showing that patients treated longer had a smaller decline in LV EF [23]. Extension of this study demonstrated that early initiation of ACEi was associated with lower mortality [24]. More recently, Porcher et al., found that prophylactic ACEi use in DMD was associated with significantly higher overall survival and lower rate of HF hospitalization when analyzing over 500 males in the French DMD Heart Registry[25]. The benefit of prophylactic mineralocorticoid receptor antagonist (MRA) use in patients with DMD has been shown in sequential studies by Raman et al. Eplerenone daily (added to a background of ACEi) was found to be superior to placebo, with eplerenone attenuating the progression of decline in cardiac contractile function by CMR, particularly for those treated at a younger age. With regards to choice of MRA, spironolactone was noninferior to eplerenone in preserving cardiac function with better stabilization of LV strain with a dose of 50 mg (compared to 25 mg) regardless of MRA used [26–28].

For the management of DMD CM with ventricular dysfunction, multiple studies have evaluated the use of combination therapy with ACEi and beta-blocker (BB). Viollet et al., studied a cohort of 42 teens with DMD, comparing ACEi therapy to ACEi plus BB. Both treatment groups showed a significant improvement in LV EF compared to pre-therapy, with no difference between the two treatment groups [29]. Murphy et al., and Kisel et al., both evaluated single-center retrospective cohorts of adults with DMD and found that long-term combination use of ACEi and BB was associated with a reduced decline in LV EF [30, 31]. There is emerging data on the use of angiotensin receptor-neprilysin inhibitor (ARNI) in the DMD population; Arcudi et al., evaluated a small cohort of DMD patients with EF < 40% treated with ARNI and showed a significant increase in EF with short-term treatment [32]. Lastly, the safety and tolerability of sodium-glucose cotransporter-2 inhibitor (SGLT2i) is being explored in the DMD population given its role in other populations of HF patients as well as promising data from a dystrophin-deficient mdx mouse model with empagliflozin treatment able to rescue abnormally reduced sodium currents of ventricular cardiomyocytes, suggesting this may diminish arrhythmia vulnerability for DMD patients [33, 34].

Despite a better understanding of the evolution of DMD CM, the optimal age of initiation of prophylactic cardiac medication and the optimal combination and target dosing of oral HF medications to manage DMD CM remains unknown. Given this, consensus recommendations for cardiac medication use for DMD CM were published by Advanced Cardiac Therapies Improving Outcomes Network (ACTION), representing DMD cardiologists from > 20 institutions, to standardize practice [35]. Additionally, the ACTION Dystrophinopathy Registry was established in part, to understand best practices with regards to oral HF medications. This study seeks to understand cardiac medication use in a large cohort of males with DMD followed prospectively in the ACTION Dystrophinopathy registry with focus on current medication practices and adherence to consensus directed medical therapy (CDMT).

## Methods

ACTION is a pediatric HF learning network, including > 50 centers, which seeks to improve critical outcomes in children and young adults with HF [36]. Data for this study was obtained by querying the ACTION Dystrophinopathy Registry. The ACTION Dystrophinopathy Registry was created in 2021 to expand our understanding of current cardiac outcomes, to increase the number of patients receiving consensus directed medical therapy (CDMT), to increase the number of patients receiving timely consideration of advanced cardiac therapies, and to understand cardiac safety and potential efficacy of gene therapy. The enrolled population includes all dystrophinopathy patients ≥ 10 years of age, any dystrophinopathy patient with evidence of CM regardless of age, and dystrophinopathy patients who have received gene therapy regardless of age. Data is entered initially at enrollment and then prospectively with a frequency of every 6 months thereafter. Data collection includes demographics, clinical information such as ambulation status, respiratory support needed, neuromuscular status and therapies used, cardiac medication use, ambulatory rhythm monitoring, pulmonary function testing, and cardiac events including HF hospitalization, internal cardioverter defibrillator (ICD) implantation, ventricular assist device (VAD) implantation, and heart transplantation.

Males with DMD with at least one follow-up data entry (at least 6 months after enrollment), inclusive of medication and cardiac imaging data, were included in this analysis. Females, patients with a history of VAD or heart transplant at the time of enrollment, and those enrolled after death were excluded from analysis. Additionally, patients who did not have cardiac imaging performed within 6 months (prior) of enrollment and most recent follow-up were excluded from analysis. Patient diagnosis was determined by the enrolling centers and cardiac function data was obtained from local reports. LV systolic dysfunction was defined as LV EF < 55%, Fractional Shortening (FS) < 28%, or qualitative assessment of LV systolic dysfunction (i.e., mild, moderate, or severely depressed). Moderate or worse dysfunction was defined as an LV EF ≤ 40%, FS < 20%, or moderate or severe dysfunction by qualitative assessment. Cardiac function data was obtained by CMR or echocardiogram, whichever was most recently done at the time of enrollment and at the time of the most recent follow-up data entry. If both were done within similar timeframe, CMR data was analyzed. For echocardiogram data, the LV EF was the preferred functional assessment. If EF was not available, FS was used. If FS was not available, qualitative assessment of LV systolic function was used. For cardiac medication use in the presence of LV systolic dysfunction, CDMT was defined as ACEi/angiotensin receptor blocker (ARB)/ARNI, plus BB, plus MRA. CDMT target doses came from the ACTION Muscular Dystrophy Committee consensus recommendations which were initially created and circulated to ACTION centers in January of 2021 and recently accepted for publication [35]. Recommendations were made for dosing targets ‘prior to’ HF with reduced ejection fraction (HFrEF) and ‘with’ HFrEF; both were analyzed.

Patients were prospectively consented and enrolled at participating centers following Institutional Review Board (IRB) approval prior to any data collection. The majority of sites rely on the central IRB at Cincinnati Children’s Hospital Medical Center. Centers not relying on Cincinnati Children’s Hospital obtain local IRB approval prior to consent and enrollment. For all ACTION registries, data quality and integrity are ensured by the data coordinating center (DCC) using internal reliability checks, regular site queries for missing or questionable data, and adjudication of select major events by an adjudication committee. Adjudicated events include all neurologic events, mortality, VAD replacement, and 15% of all other events selected at random.

## Analysis

Descriptive statistics were used to report patient characteristics and outcomes. Continuous data is presented as median (IQR, interquartile range) and categorical data is presented as frequency (percentage). Differences in medication use and CDMT between enrollment and follow-up were compared using chi-square tests, with a p-value < 0.05 considered statistically significant. Analyses were conducted in R (R Core Team, 2023).

## Results

**Fig. 1:**
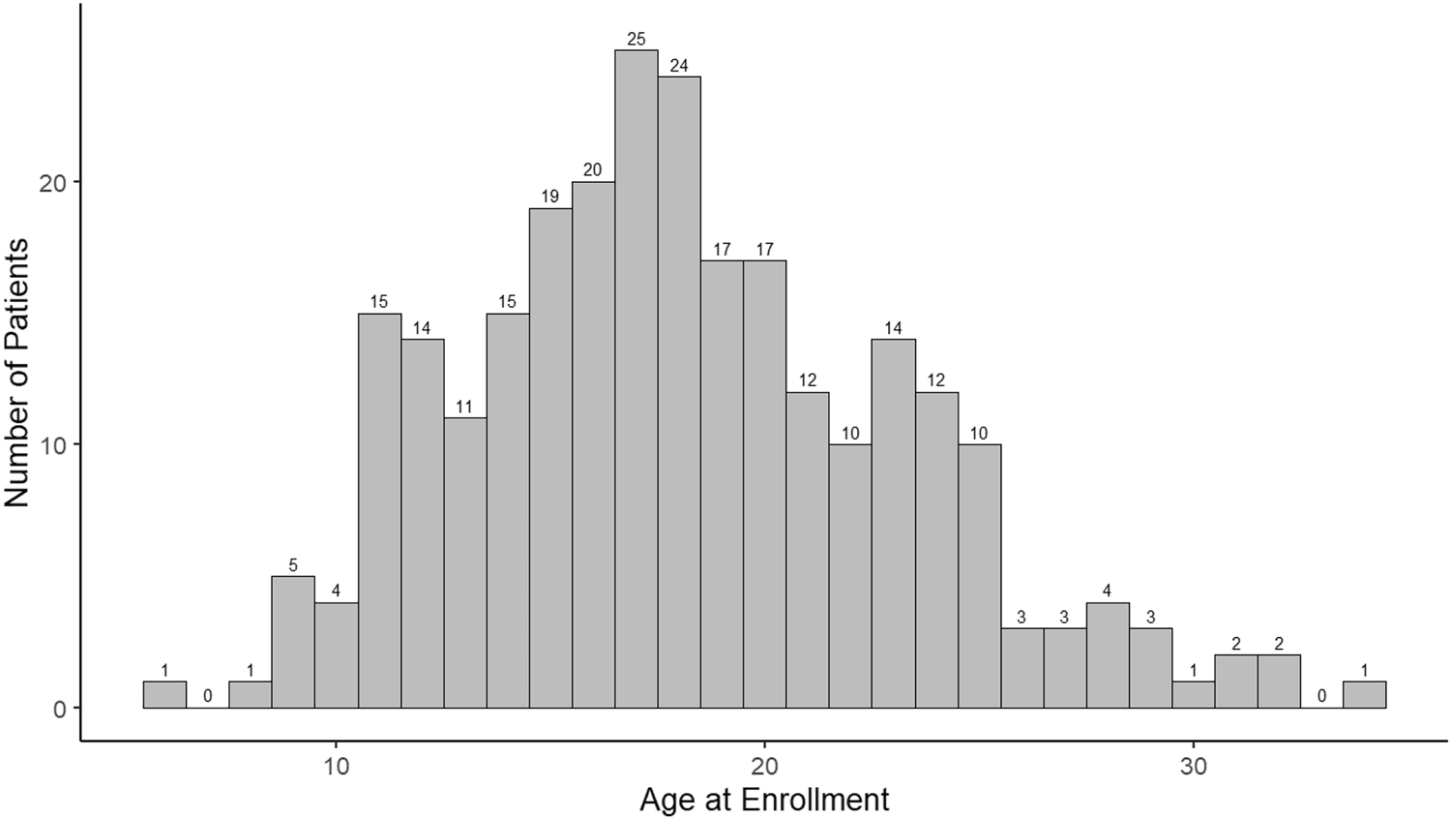
Age distribution of the 265 enrolled males with Duchenne Muscular Dystrophy

At the time of analysis, 353 patients had been enrolled in the ACTION Dystrophinopathy registry and had at least one prospective follow-up data entry. Ninety-three patients were excluded (females (N = 4), patients with Becker Muscular Dystrophy or an Intermediate/Unknown phenotype (N = 27), patients enrolled after VAD implant or heart transplant (N = 5), and patients with missing cardiac function or medication data (N = 57)) resulting in a cohort of 265 males with DMD. Patient data for this analysis was submitted by 22 centers (Supplemental Table 1) and median follow-up for this cohort (from enrollment to most recent follow-up data entry) was 11.5 (IQR 6.2–15.6, range 3–55.9) months. The median age at enrollment was 17.5 (IQR 14.5–21.5) years with the age breakdown represented in Fig. 1. Demographics are shown in Table 1; most of the cohort was white (81.1%), not Hispanic or Latinx (89.8%), non-ambulatory (76.6%), using respiratory support (54.0%), and on steroids (82.6%).

**Table 1:** Demographics of the 265 enrolled males with Duchenne Muscular Dystrophy

Demographics at Enrollment	Median (IQR) or N (%)
Age at enrollment (years)	17.5 (IQR 14.5–21.5)
Race	
White	215 (81.1%)
Black or African American	22
Asian	10
Native American/Alaska Native	2
Other	16
Ethnicity	
Not Hispanic or Latinx	238 (89.8%)
Hispanic or Latinx	27
Genetic testing results available	250 (94.3%)
Variant type	
Deletion	186 (74.4%)
Duplication	32
Point mutation	23
Other	5
Unknown	4
Ambulatory	62 (23.4%)
Age at loss of ambulation (years)	12.0 (IQR 10.0–13.0)
Currently using respiratory support	143 (54.0%)
Non-Invasive respiratory support	141 (98.6%)
BIPAP	126 (89.4%)
CPAP	8
SIP Ventilator	23
Invasive respiratory support	2 (1.4%)
Currently on steroids	219 (82.6%)
Received gene therapy	9 (3.4%)
Receiving additional neuromuscular therapies	45 (17%)
Eteplirsen	20 (44.4%)
Casimersen	10
Ataluren	8
Golodirsen	3
Vitolarsen	4

### Cardiac Function Data

At enrollment, 142 patients (53.6%) had decreased LV systolic function with 123 having normal LV systolic function. The median age at enrollment for patients with decreased LV systolic function was 18.2 (IQR 15.9–20.0) years compared to 16.5 (IQR 13.2–20.0) years for those with normal LV systolic function at enrollment (*p* < 0.001). Of those with LV dysfunction at enrollment, 51 (35.9% of those with dysfunction, 19.2% of the cohort) had moderate or severe dysfunction. The median LV EF at enrollment was 53.4% (IQR 44.0%-58.0%) by CMR and 53.0% (IQR 41.51%-60.0%) by echocardiogram. At most recent follow-up, 153 patients (57.7%) had decreased LV systolic function and 112 had normal systolic function. Of those with LV dysfunction at follow-up, 67 (43.7% of those with dysfunction, 25.3% of the cohort) had moderate or severe dysfunction. The median LV EF at follow-up was 54.8% (IQR 45.4%-59.0%) by CMR and 52.0% (IQR 40.0%-58.0%) by echocardiogram. Twenty-nine patients (10.9%) had a decline in LV systolic function (met the pre-specified definition of LV systolic dysfunction) over the study period and 18 (6.8%) had improvement in LV systolic function (met the pre-specified definition of normal LV systolic dysfunction) over the study period.

### Cardiac Medication Use

Cardiac medications used for patients with LV systolic dysfunction at enrollment and at most recent follow-up are summarized in Table 2. When analyzing individual cardiac medication used in the presence of LV systolic dysfunction, the most common cardiac medication was either an ACEi, ARB, or ARNI, used by 136 of 142 (95.6%) patients at enrollment and 142 of 153 (92.8%) patients at most recent follow-up. BB and MRA were also used by most patients with LV systolic dysfunction both at enrollment (BB 78.9%, MRA 76.8%) and most recent follow-up (BB 81.0%, MRA 85.6%). For the 29 patients who had normal LV systolic function at enrollment and abnormal LV systolic function at most recent follow-up, cardiac medication changes are summarized in Supplemental Table 2; 15 (51.7%) patients had medication doses increased (11 had one medication dose increased, 4 had two medication doses increased), 5 patients had a new cardiac medication added (3 had one medication added, 2 had two medications added), 3 patients had cardiac medications transitioned within the same class (2 beta-blocker, 1 MRA), and 3 patients had cardiac medications discontinued (1 had ACEi discontinued and 1 had both ACEi and MRA discontinued).

**Table 2:** Cardiac medication used in the presence of left ventricular systolic dysfunction

Cardiac Medication	At Enrollment (N = 142)	At Most Recent Follow-Up (N = 153)
Angiotensin-converting enzyme inhibitor (ACEi)	97 (68.3%)	95 (62.1%)
Type of ACEi		
Lisinopril	90/97 (92.8%)	91/95 (95.8%)
Enalapril	6/97 (6.2%)	3/95 (3.2%)
Perindopril	1/97 (1.0%)	1/95 (1.1%)
Dose of most used ACEi (Median (IQR))	10.0 (IQR 5.0 – 15.0) mg	10.0 (IQR 10 – 20.0) mg
Angiotensin receptor blocker (ARB)	13 (9.2%)	13 (8.5%)
Type of ARB		
Losartan	13/13 (100%)	13/13 (100%)
Dose of most used ARB	50 (IQR 25.0 – 50.0) mg	50.0 (IQR 25.0 – 75.0) mg
Angiotensin receptor-neprilysin inhibitor (ARNI)	26 (18.3%)	34 (22.2%)
Beta-blocker	112 (78.9%)	124 (81.0%)
Type of beta-blocker		
Carvedilol	64/112 (57.1%)	49/124 (39.5%)
Metoprolol	43/112 (38.4%)	71/124 (57.3%)
Atenolol	2/112 (1.8%)	1/124 (0.8%)
Nadolol	2/112 (1.8%)	2/124 (1.6%)
Sotalol	1/112 (0.9%)	1/124 (0.8%)
Dose of most used beta-blocker	18.75 (IQR 9.4 – 25.0) mg	100.0 (IQR 50.0 – 200.0) mg
Mineralocorticoid receptor antagonist (MRA)	109 (76.8%)	131 (85.6%)
Type of MRA		
Spironolactone	81/109 (74.3%)	103/131 (78.8%)
Eplerenone	28/109 (25.7%)	28/131 (21.4%)
Dose of most used MRA	25.0 (IQR 25.0 – 25.0) mg	25.0 (IQR 25.0 – 25.0) mg
Non-potassium sparing diuretic	13 (9.2%)	13 (98.5%)
Type of diuretic		
Furosemide	12/13 (92.3%)	11/13 (84.6%)
Hydrochlorothiazide	0/13 (0%)	1/13 (7.7%)
Unknown	1/13 (7.7%)	1/13 (7.7%)
Dose of most used non-potassium sparing diuretic	30.0 (IQR 17.5 – 40.0) mg	30.0 (IQR 25.0 – 35.0) mg
Sodium-glucose cotransporter 2 inhibitor (SGLT2i)	12 (8.5%)	33 (21.6%)
Type of SGLT2i		
Dapagliflozin	8/12 (66.7%)	18/33 (54.5%)
Empagliflozin	4/12 (33.3%)	14/33 (42.4%)
Digoxin	5 (3.5%)	6 (3.9%)
Ivabradine	1 (0.7%)	2 (1.3%)

**Fig. 2:**
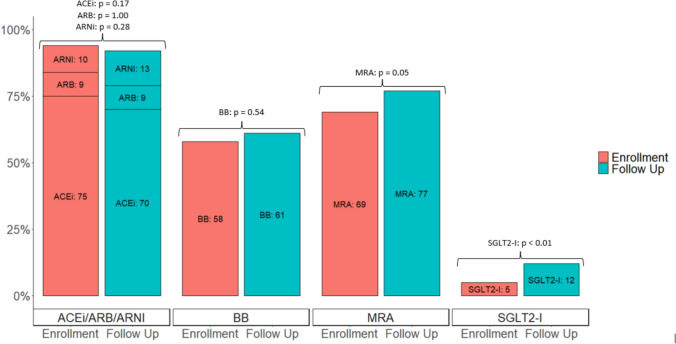
Individual cardiac medication used at enrollment and most recent follow-up for 265 males with Duchenne Muscular Dystrophy

Cardiac medications used for patients with normal LV systolic function are summarized in Table 3. Most patients with normal LV systolic function were on an ACEi or ARB at enrollment, 113 of 123 (91.9%) patients, and an ACEi, ARB, or ARNI at most recent follow-up, 101 of 112 (90.2%) patients. An MRA was also commonly used in the presence of normal LV systolic function both at enrollment and most recent follow-up, for 59.3% and 64.3% of patients, respectively. When looking at the entire cohort, irrespective of ventricular function, there were two medications with use that significantly increased or had a trend toward increased use over the follow-up period, SGLT2i and MRA, respectively (Fig. 2).

**Table 3:** Cardiac medication used in the presence of normal left ventricular systolic function

Cardiac Medication	At Enrollment (N = 123)	At Most Recent Follow-Up (N = 117)
Angiotensin-converting enzyme inhibitor (ACEi)	103 (83.7%)	90 (80.4%)
Type of ACE-I		
Enalapril	3/103 (2.9%)	4/90 (4.4%)
Lisinopril	99/103 (96.1%)	85/90 (94.4%)
Perindopril	1/103 (1.0%)	1/90 (1.1%)
Dose of most used ACEi (Median (IQR))	10.0 (IQR 5.0 – 10.0) mg	10.0 (IQR 5.0 – 10.0) mg
Angiotensin receptor blocker (ARB)	10 (8.1%)	10 (8.9%)
Type of ARB		
Losartan	10/10 (100%)	10/10 (100%)
Dose of most used ARB	25.0 (IQR 25.0 – 75.0) mg	25.0 (IQR 25.0 – 68.75) mg
Angiotensin receptor-neprilysin inhibitor (ARNI)	0 (0%)	1 (0.9%)
Beta-blocker	41 (33.3%)	37 (33.0%)
Type of beta-blocker		
Carvedilol	23/41 (56.1%)	18/37 (48.6%)
Metoprolol	18/41 (43.9%)	18/37 (48.6%)
Atenolol	0/0 (0%)	1/37 (2.7%)
Dose of most used beta-blocker	12.5 (IQR 9.38 – 25.0) mg	12.5 (IQR 12.5 – 25.0) mg
Mineralocorticoid receptor antagonist (MRA)	73 (59.35%)	72 (64.3%)
Type of MRA		
Spironolactone	55/73 (75.3%)	47/72 (65.3%)
Eplerenone	18/73 (24.7%)	25/72 (34.7%)
Dose of most used MRA	25.0 (IQR 25.0 – 25.0) mg	25.0 (IQR 25.0 – 25.0) mg
Non-potassium sparing diuretic	1 (0.7%)	0 (0%)
Type of diuretic		
Furosemide	1/1 (100%)	-

**Fig. 3:**
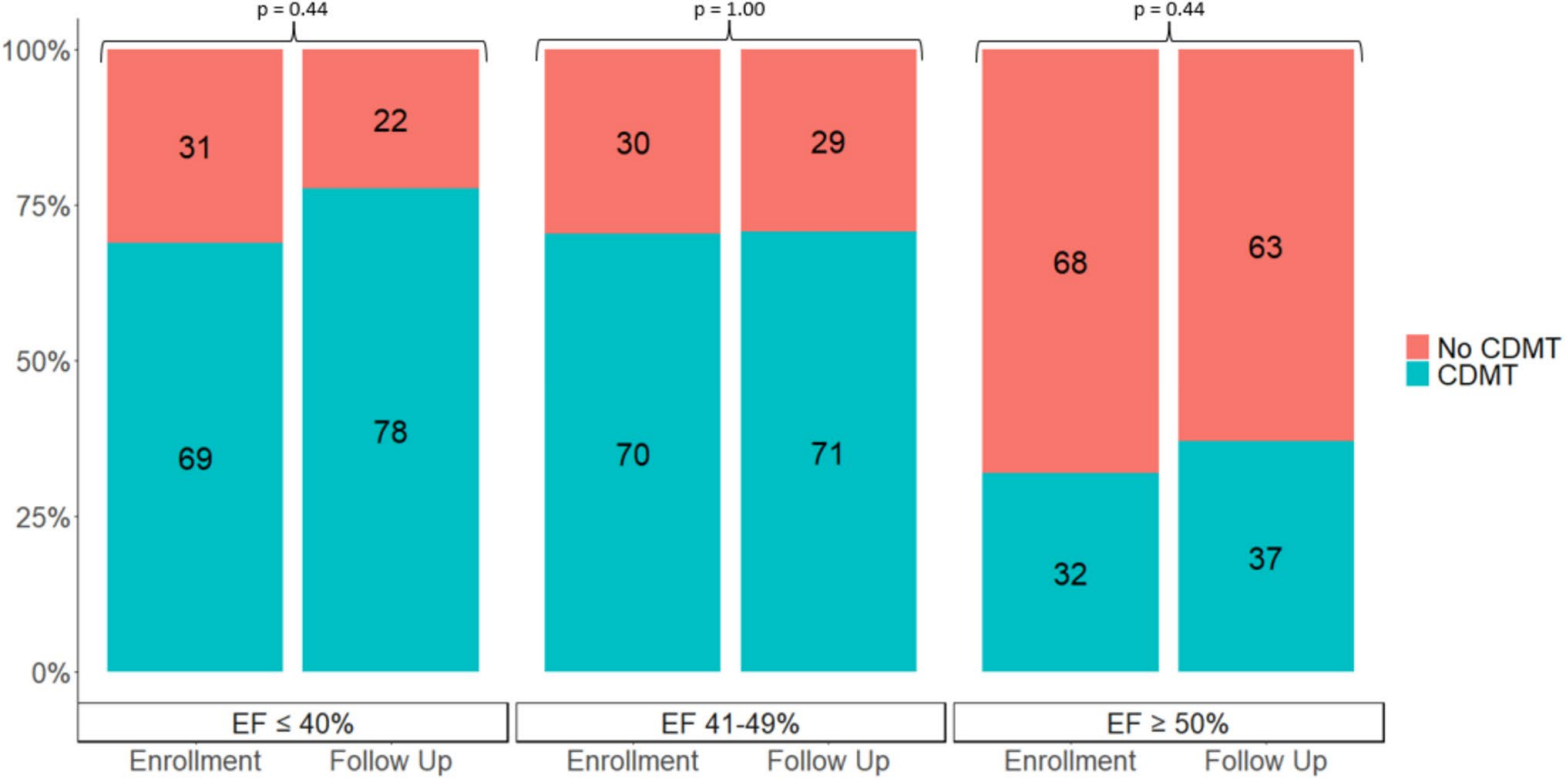
Consensus driven medical therapy at enrollment and most recent follow-up based on ejection fraction

**Fig. 4:**
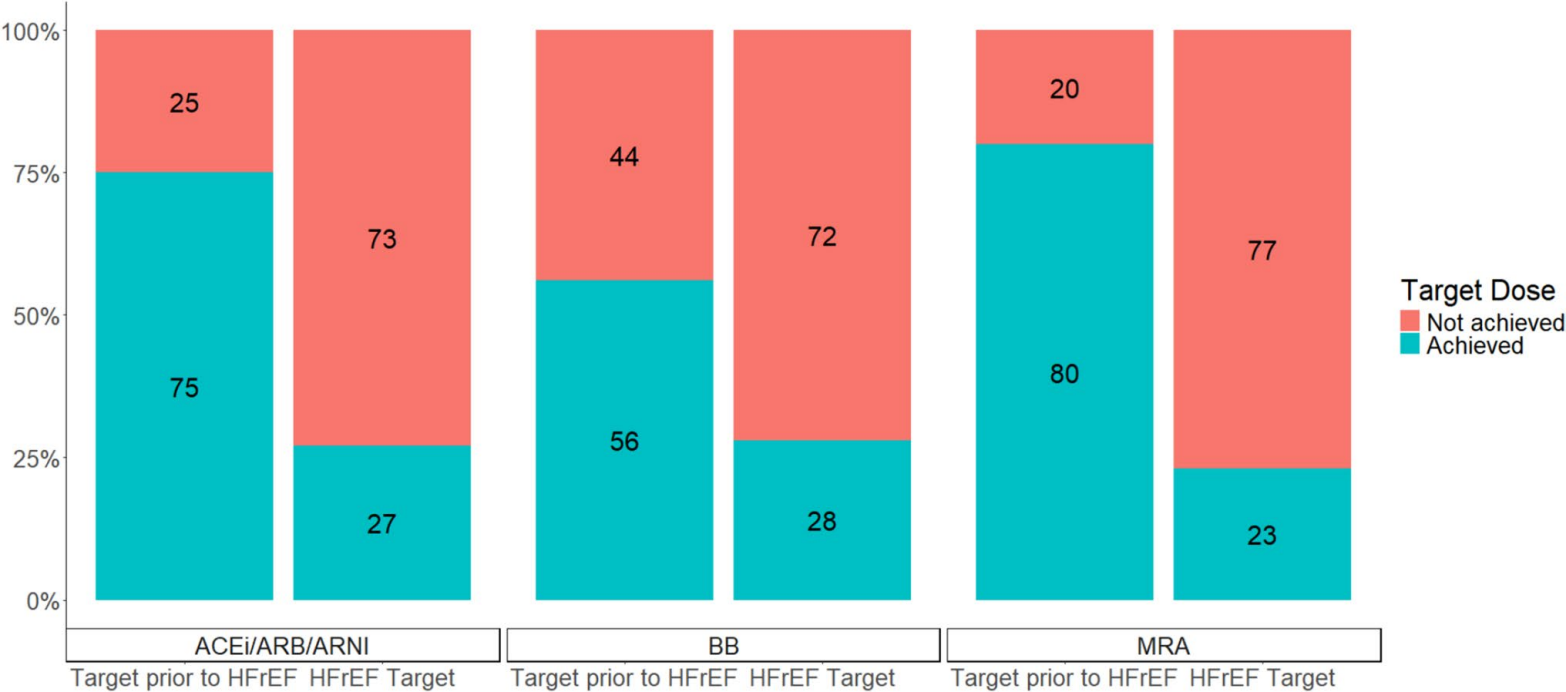
Percent of the cohort with moderate or worse left ventricular systolic function achieving target doses of consensus driven medical therapy at most recent follow-up

For patients with moderate or worse LV systolic dysfunction, CDMT was used for 36/51(70.6%) at enrollment and 49/67 (73.1%) at most recent follow-up. CDMT at enrollment and most recent follow-up based on EF (when EF was available) is shown in Fig. 3. The frequency of CDMT use by EF did not differ over the follow-up period. Target doses of CDMT (Supplemental Table 3) for patients with HFrEF (defined for this analysis as moderate or worse LV systolic function at most recent follow-up) were achieved for 26% of patients on ACEi/ARB/ARNI, 28% on BB, and 23% on MRA (Fig. 4). When using the target doses ‘prior to HRrEF,’ the target dose was achieved for 75% of patients on ACEi/ARB/ARNI, 56% on BB, and 80% on MRA.

Data on medication discontinuation is summarized in Table 4. The medication with the highest discontinuation rate was ACEi (29.7%), however, the most common reason for discontinuation of an ACEi was transition to an ARB/ARNI (16/27, 59.3%). MRA had the second highest rate of discontinuation (9.7%) with the most common indication being patient symptoms (3/6, 50.0%). BB, ARB, and SGLT2i had low rates of discontinuation (3.9%, 1.7%, and 0.4%, respectively). There were no cases of ARNI discontinuation.

**Table 4:** Cardiac medication non-use at most recent follow-up

Cardiac Medication	Number of patients not using	Number of patients previously using	Reason for discontinuation	
Angiotensin-converting enzyme inhibitor	91	27/91 (29.7%)	Transitioned to ARB/ARNI Hypotension Patient symptoms Hyperkalemia Transitioned to milrinone VAD implant Unknown	16 3 3 2 1 1 1
Angiotensin receptor blocker	242	4/242 (1.7%)	Hypotension Unknown	3 1
Beta-blocker	104	4/104(3.9%)	Patient preference Hypotension Transitioned to milrinone VAD implant	1 1 1 1
Mineralocorticoid receptor antagonist (MRA)	62	6/62 (9.7%)	Patient symptoms Hyperkalemia Sepsis VAD implant	3 1 1 1
Sodium-glucose cotransporter 2 inhibitor	232	1/232 (0.4%)	Urinary tract infection	1

### Outcomes

Acute HF hospitalizations were experienced by 12 (4.5%) patients prior to enrollment (median age at first HF hospitalization 16.2 (IQR 15.4–21) years); 8 patients had one hospitalization, 1 patient had two hospitalizations, and 3 patients had three hospitalizations. Over the follow-up period, 2 of these patients had additional HF hospitalizations; 1 patient had one additional hospitalization and 1 patient had three additional hospitalizations. Eight additional patients (7.5% of cohort experiencing an acute HF hospitalization in total) experienced their first HF hospitalization over the follow-up period for this study (median age at first HF hospitalization 19.2 (IQR 17.1–22.1) years); 4 had one hospitalization and 4 had two hospitalizations. With regards to the use of advanced cardiac therapies over the follow-up period, 3 patients had an ICD placed for non-sustained ventricular tachycardia (median age 16.1 (IQR 15.6–24.7) years)), 2 had a VAD placed (ages 9.2 and 29.5 years), and 1 patient had a heart transplant (age 9.7 years). At the end of the follow-up period, most of the cohort was alive (N = 246, 92.8%); there were 15 deaths (5.7%), 3 patients lost to follow-up, and 1 patient with an unknown status. The median age of death was 19.6 (IQR 15.8–25.3) years. Cause of death was classified as sudden cardiac death (n = 3, 20.0%), progressive heart failure (n = 3, 20.0%), infection (n = 2, 13.3%), respiratory failure (n = 1, 6.7%), multi-organ failure (n = 1, 6.7%), unknown (n = 3, 20.0%), and other (n = 2, 13.3%). The other reasons listed for cause of death were dropped during transfer with subsequent decompensation and post-operative arrest after orthopedic surgery.

## Discussion

The ACTION Dystrophinopathy Registry is the first known prospective registry of males with DMD focused on cardiac therapies and cardiac outcomes. Initial analysis of a large cohort with complete follow-up data at 1 year, showed high use of RAAS (renin–angiotensin–aldosterone system) blockade, irrespective of ventricular function, with ACEi (or ARB/ARNI) and an MRA. In the presence of moderate or severe LV dysfunction, gaps were found both in the number of patients not on recommended cardiac medications and not on recommended target doses of each of the cardiac medications.

The effectiveness of prophylactic cardiac medication use in DMD has been shown in studies evaluating both ACEi and MRA first published nearly 20 years ago, and now integrated into the most recent DMD cardiac care recommendations published in 2018 [22–24, 26–28]. With the current recommendation that an ACEi be initiated by age 10, it is reassuring to see that approximately 90% of patients with normal LV systolic function in this cohort are on an ACEi (or equivalent). Prior work by Wittlieb-Weber et al. analyzing the MDSTAR*net* registry in the setting of earlier DMD cardiac recommendations (American Academy of Pediatrics, 2005), showed that of 458 patients with a normal echocardiogram, 29.3% were prescribed a cardiac medication (most often ACEi alone) and that when comparing years 2000–2005 to years 2006–2011, patients were 2.3 times more likely to be prescribed a cardiac medication in the setting of a normal echocardiogram [37, 38]. The evolution in practice can likely be explained by more data to support the prophylactic use of ACEi in DMD as well as by more clarity in published consensus recommendations. Mejia et al., used the IBM MarketScan Research Database to evaluate cardiac specialty care for children with muscular dystrophy in the United States, and found a low proportion of days covered > 80% for ACEi/ARB (13.6%) among patients in the 10–18 year age group [39]. Though it is challenging to understand the striking difference in findings, different methodology was used for these analyses, and it should be noted that patients analyzed in the present study include patients followed at tertiary care centers with specific expertise in DMD cardiac management.

Though most of the cohort with normal LV systolic function was on a MRA at most recent follow-up (64.3%) and MRA use increased over the follow-up period, irrespective of ventricular function (from 69 to 77%, *p* = 0.05), prophylactic use was not as high as it was for ACEi. Villa et al., surveyed pediatric cardiology providers within ACTION to analyze current practices in treating DMD CM and found variability in practice with regards to MRA initiation. Though the majority of providers surveyed (28/31, 90%) routinely prescribed MRA, indications varied; 9 initiated with fibrosis found on CMR, 9 initiated with either fibrosis on CMR or presence of systolic dysfunction, 7 initiated solely based on the presence of systolic dysfunction, and 3 initiated prophylactically at age 10 [40]. The DMD Care Considerations (2018) made no comment on the prophylactic use of MRA. The ACTION Muscular Dystrophy Committee consensus recommendations for DMD cardiac treatment, state that it is reasonable to initiate MRA in patients with fibrofatty changes (by CMR) at any age or by 12 years of age, given this is the average age of first detection of LGE (late gadolinium enhancement) on CMR [35]. With clarity in consensus recommendations regarding MRA, it is likely use will increase further over time.

CDMT was used for approximately 70.0% of DMD patients with moderate or severe LV systolic dysfunction, with no significant change over the follow-up period. Though this rate of CDMT use can be viewed favorably as it is higher than previously reported in an analysis of DMD patients with systolic dysfunction on cardiac medications, it leaves a notable gap for vulnerable patients (i.e., those with the worst cardiac function) [38]. It is important to acknowledge that the term ‘consensus’ directed is being used in place of ‘guideline’ directed for medical therapy targets. This terminology was chosen given that published adult HF guidelines are based on decades of HF studies in patients with reduced systolic function not inclusive of patients with dystrophic CM [19, 41]. Though some data exists on the use of HF medications for reduced systolic function in DMD CM, the medication recommendations evaluated in this study come from consensus expert opinion [35]. Deficits in adherence to HF medication guidelines is not unique to the DMD population as it is well documented in both the pediatric and adult HF literature across the world, that adherence to HF guidelines for patients with reduced EF is not optimal both with initiation of each cardiac medication and ability to achieve target dosing [42–45]. DMD patients are unique in the complexity of care coordination required with the need to follow serially with many providers in addition to cardiology, including neuromuscular, pulmonary, endocrinology, and physical therapy, which may be a barrier to achieving the goal cardiac care plan[22, 46]. Additionally, tolerance of cardiac medications that can lower blood pressure is an issue for DMD patients who have lower blood pressure at baseline and this likely limits achieving target dosing; approximately 20% of patients analyzed in this study who were previously on ACEi or ARB discontinued the medication due to hypotension. Further, most patients with HFrEF did achieve the dosing target ‘prior to HFrEF’ which implies they were able to tolerate lower doses of all 3 classes of medications. This suggests we may need to alter blood pressure targets for these patients (in the absence of symptoms), adjust our dosing targets for this population, or focus more on understanding the role of cardiac medications with less hemodynamic effect such as SGLT2i. The use of SGLT2i did significantly increase over the follow-up period for this cohort, though, use remained low (12%); more research is needed to understand the benefit of this newer class of HF medication for patients with dystrophic CM. With data showing that a higher dose of MRA (50 mg) had better stabilization of LV strain compared to a lower dose (25 mg), this seems to be an achievable target of intervention (as only 23% of patients achieved this dose) given limited hemodynamic effect, though appropriate lab surveillance is necessary [28]. The importance of the multidisciplinary clinic for DMD patients cannot be overstated as this is crucial to bring together all the needed care for these patients to ensure compliance with recommended therapies across the specialties. Ensuring all DMD patients have access to such a resource either in-person or through telemedicine care is key future work.

The burden of cardiac disease for DMD patients is highlighted in this large, prospectively followed cohort. Of the 265 males with DMD analyzed, 142 (53.6%) had ventricular dysfunction at the time of enrollment, 20 (7.5%) had an acute HF hospitalization, and 15 (5.7%) died over the approximate one year of follow-up. Death occurred at a median of 19.6 (IQR 15.8–25.3) years with cause of death being cardiac in nature in 6 cases (sudden cardiac death = 3, progressive HF = 6); notably cause of death was unknown for 3 (20%) patients and so it is possible that more deaths were cardiac in origin. Given our knowledge that CM is nearly universal in DMD patients as they age, as shown by Nigro et al., it is imperative that we take more of a proactive rather than a reactive approach to delay the onset and progression of cardiac disease[5]. The best age to initiate prophylactic cardiac medication has yet to be determined, and one could argue, the earlier the better particularly when considering medications with some proven benefit and a favorable safety profile. Further, the benefit of combination prophylactic therapy warrants further study particularly when considering the possible cardiac effects of recent Food and Drug Administration (FDA) approved neuromuscular therapies such as delandistrogene moxeparvovec, givinostat, and vamorolone [47–49]. Lastly, understanding the optimal combination of cardiac medications, along with optimal doses, in the setting of LV systolic dysfunction for patients with dystrophic CM is incompletely understood. It is therefore imperative to continue to follow DMD patients in a prospective way with targeted cardiac data points in mind [47–49]. This was a key purpose in the creation of the ACTION Dystrophinopathy registry and supports ongoing efforts to enroll patients.

There are limitations of this study to mention. As with any registry analysis, incomplete data is a possibility as is loss to follow-up for patients that may transition care or not return for recommended care. We limited analysis to patients with complete enrollment and follow-up forms which were requested approximately every 6 months; completeness of follow-up data entry did vary by center and did narrow our cohort. Medication use was pulled from chart review and determined based on clinic notes and the medication record. Adherence or non-adherence was only able to be assessed by chart review and not cross checked with pharmacy data and so, it is possible that what a patient was prescribed and what they were taking could differ. Medication dosing changes that were not completely documented or that occurred immediately after a data entry, would have been missed. Similarly, medication side effect data came from chart review as well and the reason for medication discontinuation was best assessed at the time of data extraction and as such, brief discontinuation of a medication may not have been accounted for. This study did not assess sociodemographic factors that may impact cardiac care, nor did it evaluate patient or caregiver reported outcomes with regards to perception of cardiac medication use or burden of medication use which is important to consider for patients with such a complex, chronic medical condition as DMD. Lastly, this analysis includes patients enrolled in a specialized registry who are predominantly followed at tertiary care centers with DMD expertise and the follow-up period was short. Data reported may overrepresent a DMD population with higher access to specialty cardiac care and underestimate barriers experienced by many patients with DMD. It is likely that medication practices will continue to evolve over longer-term follow-up.

## Conclusion

Initial analysis of a large, prospective registry of males with DMD showed that 30% of patients with moderate or severe LV dysfunction were not on CDMT and those that were, most often did not reach suggested target dosing. Though it is important to better understand the optimal combination and target dosing of cardiac medications for DMD CM and the barriers to optimization of CDMT given the increasing frequency of cardiac causes of death for DMD patients, more emphasis needs to be placed on proactive rather than reactive cardiac care. Further study of the optimal combination of prophylactic cardiac medications and the ideal age of initiation is key to delaying the onset and progression of DMD CM particularly in the era of novel neuromuscular therapies. Registries such as the ACTION Dystrophinopathy registry are crucial in our ability to alter the trajectory of cardiac disease for DMD patients and further, may allow us to establish a control group with regards to baseline CDMT for purposes of future prospective cardiac drug trials in the DMD population.

## Supplementary Information

Below is the link to the electronic supplementary material.

Supplementary file1 (DOCX 22 KB)

Supplementary file2 (DOCX 32 KB)

## Data Availability

Data is provided within the manuscript or supplementary information files.
