# Supplementary material for: Cardiac Medication Use in ACTION for Duchenne Muscular Dystrophy Cardiomyopathy
Source: Pediatr Cardiol. 2025 Jun 20;47(4):1439–50. doi: 10.1007/s00246-025-03917-2 (PMC12945980; doi:10.1007/s00246-025-03917-2)
Supplement: Supplementary file 1 — Supplementary file1 (DOCX 22 KB) [file 246_2025_3917_MOESM1_ESM.docx]

**Supplemental Tables.**

Supplemental Table 1. Cardiac medication management for the 29 patients with a decline in left ventricular systolic function over the follow-up period.

|  | Enrollment EF / FS / Qualitative Function | Follow-up EF / FS / Qualitative Function | Enrollment Medications  (Dose/Frequency) | Medications Stopped During Follow-up | Medications Started During Follow-Up  (Dose) | Dose Change During Follow-up  (New Dose / New Frequency) |
| --- | --- | --- | --- | --- | --- | --- |
| Patient 1 | EF 56.0% | EF 50.0% | ARB: Losartan  (150 mg daily)  BB: Carvedilol  (50 mg BID)  MRA: Spironolactone (25 mg daily) | BB: Carvedilol | BB: Metoprolol (150 mg daily) | N/A |
| Patient 2 | EF 58.0% | EF: 51.0% | ACE: Lisinopril  (10 mg daily)  BB: Metoprolol  (100 mg daily)  MRA: Spironolactone (25 mg daily) | N/A | N/A | BB: Metoprolol (200 mg daily) |
| Patient 3 | FS 41.1% | EF: 50.0% | BB: Metoprolol  (100 mg daily)  MRA: Spironolactone (25 mg daily) | N/A | N/A | N/A |
| Patient 4 | EF 68.0% | EF: 45.8% | ACE: Lisinopril  (15 mg daily)  BB: Metoprolol  (200 mg daily)  MRA: Spironolactone (25 mg daily) | N/A | SGLT2: Dapagliflozin (10 mg daily) | N/A |
| Patient 5 | EF 69.0% | EF 54.0% | MRA: Spironolactone (25 mg daily)  Diuretics: Furosemide | Diuretics:  Furosemide | ACE: Lisinopril (10 mg daily) | N/A |
| Patient 6 | EF 55.0% | EF 53.0% | ACE: Lisinopril  (20 mg daily)  BB: Metoprolol  (100 mg daily)  MRA: Spironolactone (25 mg daily) | N/A | N/A | N/A |
| Patient 7 | EF 58.0% | EF 51.0% | ACE: Lisinopril  (20 mg daily)  BB: Carvedilol  (25 mg BID)  MRA: Spironolactone (25 mg daily) | N/A | N/A | N/A |
| Patient 8 | EF 63.0% | EF 39.9% | ACE: Lisinopril  (20 mg daily)  BB: Metoprolol  (100 mg daily)  MRA: Spironolactone (25 mg daily) | N/A | N/A | BB: Metoprolol (150 mg daily) |
| Patient 9 | EF 55.0% | EF 43.0% | ACE: Enalapril  (10 mg BID)  BB: Metoprolol  (150 mg daily)  MRA: Spironolactone (25 mg daily) | N/A | N/A | BB: Metoprolol (200 mg daily) |
| Patient 10 | EF 56.0% | EF 51.0% | ACE: Lisinopril  (15 mg daily)  BB: Carvedilol  (25 mg BID)  MRA: Spironolactone (25 mg daily) | N/A | N/A | N/A |
| Patient 11 | EF 69.0% | EF 54.0% | ACE: Lisinopril  (10 mg daily)  BB: Carvedilol  (12.5 mg BID)  MRA: Spironolactone (25 mg daily) | N/A | N/A | BB: Carvedilol  (25 mg BID) |
| Patient 12 | EF 62.0% | EF 49.0% | ACE: Perindopril  (4 mg daily)  BB: Carvedilol  (25 mg BID) | N/A | N/A | N/A |
| Patient 13 | EF 56.0% | EF 53.0% | ACE: Lisinopril  (5 mg daily) | N/A | BB: Metoprolol (3.125 mg BID)  MRA: Spironolactone (25 mg daily) | ACE: Lisinopril (10 mg daily) |
| Patient 14 | Normal | FS 26.0% | ACE: Lisinopril  (5 mg daily) | N/A | N/A | N/A |
| Patient 15 | EF 60.0% | EF 46.0% | ACE: Lisinopril  (5 mg daily) | N/A | BB: Carvedilol (6.25 mg twice per day)  MRA: Spironolactone (25 mg daily) | ACE: Lisinopril (10 mg daily) |
| Patient 16 | EF 60.0 | EF 42.0 | ACE: Lisinopril  (10 mg daily)  BB: Metoprolol  (50 mg BID)  MRA: Spironolactone (25 mg daily) | N/A | N/A | ACE: Lisinopril (15 mg daily)  BB: Metoprolol (150 mg daily) |
| Patient 17 | FS 34.0% | EF 53.0% | ACE: Lisinopril  (10 mg daily)  BB: Carvedilol  (6.25 mg TID) | N/A | N/A | N/A |
| Patient 18 | FS 31.6% | EF 52.6% | ACE: Lisinopril  (10 mg daily)  BB: Carvedilol  (12.5 mg BID)  MRA: Eplerenone  (50 mg daily) | MRA: Eplerenone | MRA: Spironolactone (50 mg daily) | ACE: Lisinopril  (20 mg daily)  BB: Carvedilol (18.75 mg BID) |
| Patient 19 | EF 56.2% | EF 52.4% | ACE: Lisinopril  (7.5 mg daily)  BB: Carvedilol  (6.25 mg daily)  MRA: Spironolactone (50 mg daily) | BB: Carvedilol | BB: Metoprolol  Diuretics: Furosemide | N/A |
| Patient 20 | EF 59.0% | EF 52.0% | ACE: Lisinopril  (5 mg daily)  BB: Carvedilol  (9.375 mg BID)  MRA: Spironolactone (50 mg daily) | N/A | N/A | ACE: Lisinopril (10 mg daily)  BB: Carvedilol (12.5 mg BID) |
| Patient 21 | EF 65.0% | EF 53.4% | ACE: Lisinopril  (7.5 mg daily)  BB: Carvedilol  (9.375 mg BID)  MRA: Eplerenone  (50 mg daily) | N/A | N/A | ACE: Lisinopril (10 mg daily) |
| Patient 22 | EF 56.0% | EF 51.4% | ACE: Lisinopril  (10 mg daily)  BB: Carvedilol  (6.25 mg BID)  MRA: Spironolactone (50 mg daily) | N/A | N/A | N/A |
| Patient 23 | EF 58.0% | EF 52.8% | ACE: Lisinopril  (7.5 mg daily)  MRA: Eplerenone  (25 mg daily) | N/A | BB: Metoprolol (25 mg daily) | MRA: Eplerenone  (50 mg daily) |
| Patient 24 | EF 66.0% | EF 52.0% | ARB: Losartan  (25 mg daily)  BB: Metoprolol  (25 mg daily)  MRA: Spironolactone (25 mg daily) | N/A | N/A | ARB: Losartan (100 mg daily)  BB: Metoprolol (100 mg daily) |
| Patient 25 | EF 55.1% | EF 39.0% | ACE: Lisinopril  (10 mg daily) | N/A | MRA: Eplerenone  (25 mg daily) | ACE: Lisinopril (20 mg daily) |
| Patient 26 | EF 58.0% | EF 54.2% | ACE: Lisinopril  (10 mg daily)  MRA: Spironolactone (25 mg daily) | N/A | BB: Carvedilol (18.75 mg BID) | N/A |
| Patient 27 | EF 55.0% | EF 45.0% | ACE: Lisinopril  (2.5 mg daily) | N/A | N/A | N/A |
| Patient 28 | EF 66.0% | EF 50.0% | ARB: Losartan  (25 mg daily) | N/A | N/A | N/A |
| Patient 29 | EF 69.0% | EF 52.0% | ACE: Lisinopril  (10 mg daily) | N/A | BB: Metoprolol (12.5 mg daily) | N/A |

Supplemental Table 2. Target cardiac medication doses for analysis of consensus driven medical therapy based on ACTION consensus recommendations.

| Cardiac Medication | Target prior to HFrEF | HFrEF Target |
| --- | --- | --- |
| Enalapril | 5 mg twice per day | 10 mg twice per day |
| Lisinopril | 10 mg daily | 20 mg daily |
| Losartan | 100 mg daily | 150 mg daily |
| Sacubitril-valsartan | 24/26 mg twice per day* | 97/103 mg twice per day |
| Carvedilol | 12.5 mg twice per day | 25 mg twice per day |
| Metoprolol | 100 mg daily | 200 mg daily |
| Eplerenone | 25 mg daily | 50 mg daily |
| Spironolactone | 25 mg daily | 50 mg daily |

HFreEF= heart failure with reduced ejection fraction

*ACTION consensus recommendations did not specify a target for sacubitril-valsartan prior to HFrEF. The starting dose was used in this case.
