# Supplementary material for: Cardiac Medication Use in ACTION for Duchenne Muscular Dystrophy Cardiomyopathy
Source: Pediatr Cardiol. 2025 Jun 20;47(4):1439–50. doi: 10.1007/s00246-025-03917-2 (PMC12945980; doi:10.1007/s00246-025-03917-2)
Supplement: Supplementary file 2 — Supplementary file2 (DOCX 32 KB) [file 246_2025_3917_MOESM2_ESM.docx]

**Supplemental Tables.**

Supplemental Table 1. Participating ACTION Centers.

| **Participating ACTION Centers (N = 22, alphabetical order)** |
| --- |
| Ann & Robert H. Lurie Children’s Hospital of Chicago  Arkansas Children’s Hospital  Boston Children’s Hospital  Children’s Hospital at Montefiore  Children’s Hospital of Philadelphia  Children’s Hospital of Richmond at VCU  Children’s Medical Center at Dallas  Children’s Mercy Kansas City  Cincinnati Children’s Hospital  Dell Children’s Medical Center  Joe DiMaggio Children’s Hospital  Le Bonheur Children’s Hospital  Lucile Packard Children’s Hospital Stanford  Monroe Carell Jr. Children’s Hospital at Vanderbilt  Nationwide Children’s Hospital  Norton Children’s Hospital, University of Louisville  Phoenix Children’s Hospital  Primary Children’s Hospital  Riley Hospital for Children at IU Health  Shands Children’s Hospital  St. Louis Children’s Hospital  Stollery Children’s Hospital |

Supplemental Table 2. Cardiac medication management for the 29 patients with a decline in left ventricular systolic function over the follow-up period. Those who had a decline in left ventricular ejection fraction ≥10% are highlighted in bold.

|  | **Enrollment EF / FS / Qualitative Function** | **Follow-up**  **EF / FS / Qualitative Function**  ***Time from Enrollment EF to Follow-up EF*** | **Enrollment Medications**  **(Dose/Frequency)** | **Medications Stopped During Follow-up** | **Medications Started During Follow-up**  **(Dose)** | **Dose Change During**  **Follow-up**  **(New Dose / Frequency)** |
| --- | --- | --- | --- | --- | --- | --- |
| **Patient 1** | **EF 56.0%** | **EF 48.0%**  ***10.8 mos*** | **ACE: Lisinopril**  **(10 mg Daily)**  **BB: Carvedilol**  **(25 mg BID)**  **MRA: Spironolactone**  **(25 mg Daily)** | **N/A** | **N/A** | **N/A** |
| **Patient 2** | **EF 69.0%** | **EF 53.1%**  ***6.2 mos*** | **BB: Metoprolol XL**  **(100 mg Daily)**  **MRA: Spironolactone**  **(25 mg Daily)** | **N/A** | **N/A** | **N/A** |
| Patient 3 | EF 56.0% | EF 51.0%  *6.4 mos* | ARB: Losartan  (75 mg Daily)  BB: Carvedilol  (25 mg BID)  MRA: Spironolactone  (25 mg Daily) | N/A | N/A | N/A |
| **Patient 4** | **EF 58.0%** | **EF 50.0%**  ***11.9 mos*** | **ACE: Lisinopril**  **(15 mg Daily)**  **BB: Carvedilol**  **(25 mg BID)**  **MRA: Spironolactone**  **(25 mg Daily)** | **N/A** | **N/A** | **N/A** |
| Patient 5 | EF 55.0% | EF 52.0%  *6.0 mos* | ACE: Lisinopril  (5 mg Daily)  BB: Carvedilol  (25 mg BID)  MRA: Spironolactone  (25 mg Daily) | N/A | N/A | ACE: Lisinopril  (10 mg Daily) |
| Patient 6 | EF 57.0% | EF 53.0%  *12.2 mos* | ACE: Lisinopril  (5 mg Daily)  BB: Carvedilol  (25 mg BID)  MRA: Spironolactone  (25 mg Daily) | ACE: Lisinopril  BB: Carvedilol | BB: Metoprolol XL  (100 mg Daily) | N/A |
| **Patient 7** | **EF 66.0%** | **EF 53.0%**  ***6.2 mos*** | **ACE: Lisinopril**  **(10 mg Daily)**  **MRA: Spironolactone**  **(25 mg BID)** | **N/A** | **N/A** | **ACE: Lisinopril**  **(15 mg Daily)** |
| **Patient 8** | **EF 56.0%** | **EF 44.0%**  ***5.8 mos*** | **ACE: Lisinopril**  **(20 mg Daily)**  **BB: Metoprolol XL**  **(150 mg Daily)**  **MRA: Spironolactone**  **(25 mg Daily)** | **N/A** | **N/A** | **BB: Metoprolol XL**  **(200 mg Daily)** |
| **Patient 9** | **FS 30.0%** | **EF 47.0%**  ***19.1 mos*** | **ACE: Lisinopril**  **(10 mg Daily)**  **BB: Metoprolol XL**  **(100 mg Daily)**  **MRA: Spironolactone**  **(25 mg Daily)** | **N/A** | **N/A** | **ACE: Lisinopril**  **(15 mg Daily)**  **BB: Metoprolol XL**  **(200 mg Daily)** |
| **Patient 10** | **EF 63.0%** | **EF 40.0%**  ***5.8 mos*** | **ACE: Lisinopril**  **(20 mg Daily)**  **BB: Metoprolol XL**  **(100 mg Daily)**  **MRA: Spironolactone**  **(25 mg Daily)** | **N/A** | **N/A** | **BB: Metoprolol XL**  **(150 mg Daily)** |
| Patient 11 | EF 55.0% | EF 52.0%  *6.2 mos* | ACE: Enalapril  (10 mg BID)  BB: Metoprolol XL  (150 mg Daily)  MRA: Spironolactone  (25 mg Daily) | N/A | N/A | BB: Metoprolol XL  (200 mg Daily) |
| Patient 12 | EF 56.0% | EF 53.0%  *7.6 mos* | ACE: Lisinopril  (5 mg Daily) | N/A | BB: Carvedilol  (3.125 mg BID)  MRA: Spironolactone  (25 mg Daily) | ACE: Lisinopril  (10 mg Daily) |
| Patient 13 | Normal Function | FS 26.0%  *16.2 mos* | ACE: Lisinopril  (5 mg Daily) | N/A | N/A | N/A |
| **Patient 14** | **EF 60.0%** | **EF 46.0%**  ***60.8 mos*** | **ACE: Lisinopril**  **(5 mg Daily)** | **N/A** | **BB: Carvedilol**  **(6.25 mg BID)**  **MRA: Spironolactone**  **(25 mg Daily)** | **ACE: Lisinopril**  **(10 mg Daily)** |
| **Patient 15** | **EF 56.0%** | **EF 39.0%**  ***15.9 mos*** | **ACE: Lisinopril**  **(10 mg Daily)**  **BB: Metoprolol**  **(50 mg BID)**  **MRA: Eplerenone**  **(25 mg Daily)** | **ACE: Lisinopril**  **MRA: Eplerenone** | **N/A** | **BB: Metoprolol XL**  **(50 mg Daily)** |
| **Patient 16** | **EF 60.0%** | **EF 42.0%**  ***28.8 mos*** | **ACE: Lisinopril**  **(10 mg Daily)**  **BB: Metoprolol XL**  **(50 mg BID)**  **MRA: Spironolactone**  **(25 mg Daily)** | **N/A** | **N/A** | **ACE: Lisinopril**  **(15 mg Daily)**  **BB: Metoprolol XL**  **(150 mg Daily)** |
| **Patient 17** | **FS 34.0%** | **EF 53.0%**  ***15.2 mos*** | **ACE: Lisinopril**  **(10 mg Daily)**  **BB: Carvedilol**  **(6.25 mg TID)** | **N/A** | **N/A** | **BB: Carvedilol**  **(6.25 mg BID)** |
| **Patient 18** | **FS 31.6%** | **EF 52.6%**  ***12.7 mos*** | **ACE: Lisinopril**  **(10 mg Daily)**  **BB: Carvedilol**  **(12.5 mg BID)**  **MRA: Eplerenone**  **(50 mg Daily)** | **MRA: Eplerenone** | **MRA: Spironolactone**  **(50 mg Daily)** | **ACE: Lisinopril**  **(20 mg Daily)**  **BB: Carvedilol**  **(18.75 mg BID)** |
| Patient 19 | EF 56.0% | EF 52.4%  *12.4 mos* | ACE: Lisinopril  (7.5 mg Daily)  BB: Carvedilol  (6.25 mg Daily)  MRA: Spironolactone  (50 mg Daily) | BB: Carvedilol | BB: Metoprolol  (N/A dose/frequency) | N/A |
| **Patient 20** | **EF 59.0%** | **EF 52.0%**  ***18.0 mos*** | **ACE: Lisinopril**  **(5 mg Daily)**  **BB: Carvedilol**  **(9.375 mg BID)**  **MRA: Spironolactone**  **(50 mg Daily)** | **N/A** | **N/A** | **ACE: Lisinopril**  **(10 mg Daily)**  **BB: Carvedilol**  **(12.5 mg BID)** |
| **Patient 21** | **EF 65.0%** | **EF 53.4%**  ***10.1 mos*** | **ACE: Lisinopril**  **(7.5 mg Daily)**  **BB: Carvedilol**  **(9.375 mg BID)**  **MRA: Eplerenone**  **(50 mg Daily)** | **N/A** | **N/A** | **ACE: Lisinopril**  **(10 mg Daily)** |
| Patient 22 | EF 56.0% | EF 51.4%  *11.5 mos* | ACE: Lisinopril  (10 mg Daily)  BB: Carvedilol  (6.25 mg BID)  MRA: Spironolactone  (50 mg Daily) | N/A | N/A | N/A |
| Patient 23 | EF 58.0% | EF 52.8%  *6.2 mos* | ACE: Lisinopril  (7.5 mg Daily)  MRA: Eplerenone  (25 mg Daily) | N/A | BB: Metoprolol XL  (25 mg Daily) | MRA: Eplerenone  (50 mg Daily) |
| **Patient 24** | **EF 66.0%** | **EF 52.0%**  ***18.2 mos*** | **ARB: Losartan**  **(25 mg Daily)**  **BB: Metoprolol XL**  **(25 mg Daily)**  **MRA: Spironolactone**  **(25 mg Daily)** | **N/A** | **N/A** | **ARB: Losartan**  **(50 mg Daily)**  **BB: Metoprolol XL**  **(100 mg Daily)** |
| **Patient 25** | **EF 55.1%** | **EF 39.0%**  ***34.8 mos*** | **ACE: Lisinopril**  **(10 mg Daily)** | **N/A** | **MRA: Eplerenone**  **(25 mg Daily)** | **ACE: Lisinopril**  **(20 mg Daily)** |
| **Patient 26** | **EF 64.6%** | **EF 54.3%**  ***12.0 mos*** | **ACE: Lisinopril**  **(5 mg Daily)** | **ACE: Lisinopril** | **N/A** | **N/A** |
| Patient 27 | EF 58.0% | EF 54.2%  *20.8 mos* | ACE: Lisinopril  (10 mg Daily)  MRA: Spironolactone  (25 mg Daily) | N/A | BB: Carvedilol  (18.75 mg BID) | N/A |
| **Patient 28** | **EF 55.0%** | **EF 45.0%**  ***6.0 mos*** | **ACE: Lisinopril**  **(2.5 mg Daily)** | **N/A** | **N/A** | **N/A** |
| **Patient 29** | **EF 66.0%** | **EF 50.0%**  ***12.2 mos*** | **ARB: Losartan**  **(25 mg Daily)** | **N/A** | **N/A** | **N/A** |

Supplemental Table 3. Target cardiac medication doses for analysis of consensus driven medical therapy based on ACTION consensus recommendations.

| Cardiac Medication | Weight | Target prior to HFrEF | HFrEF Target |
| --- | --- | --- | --- |
| Enalapril | <50 kg  ≥50 kg | 0.1 mg/kg twice per day  5 mg twice per day | 0.2 mg/kg twice per day  10 mg twice per day |
| Lisinopril | <50 kg  ≥50 kg | 0.2 mg/kg daily  10 mg daily | 0.4 mg/kg daily  20 mg daily |
| Losartan | <50 kg  ≥50 kg | 0.7 mg/kg daily  100 mg daily | 1.5 mg/kg daily  150 mg daily |
| Sacubitril-valsartan | <50 kg  ≥50 kg | 24/26 mg twice per day* | 97/103 mg twice per day |
| Carvedilol | <50 kg  ≥50 kg | 0.25 mg/kg twice per day  12.5 mg twice per day | 0.5 mg/kg twice per day  25 mg twice per day |
| Metoprolol tartrate  Metoprolol succinate | <50 kg  ≥50 kg | 0.5 mg/kg twice per day  100 mg daily | 1 mg/kg twice per day  200 mg daily |
| Eplerenone | ≥25 kg | 25 mg daily | 50 mg daily |
| Spironolactone | <50 kg  ≥50 kg | 0.5-1 mg/kg daily  12.5-25 mg daily^ | 0.5-1 mg/kg daily  25-50 mg daily^ |

HFreEF= heart failure with reduced ejection fraction

*ACTION consensus recommendations did not specify a target for sacubitril-valsartan prior to HFrEF. The starting dose was used in this case.

^A dosing range was provided in the ACTION consensus guidelines. For data analysis, 25 mg daily was used for the ‘Target prior to HFrEF’ and 50 mg daily was used for ‘HFrEF Target’.
